# Exploring time series of hyperspectral images for cold water coral stress response analysis

**DOI:** 10.1371/journal.pone.0272408

**Published:** 2022-08-08

**Authors:** Daniel Langenkämper, Aksel Alstad Mogstad, Ingrid Myrnes Hansen, Thierry Baussant, Øystein Bergsagel, Ingunn Nilssen, Tone Karin Frost, Tim Wilhelm Nattkemper

**Affiliations:** 1 Biodata Mining Group, Bielefeld University, Bielefeld, Germany; 2 Ecotone AS, Trondheim, Norway; 3 NORCE Norwegian Research Centre, Randaberg, Norway; 4 Equinor ASA, Research and Technology, Trondheim, Norway; University of Rhode Island, UNITED STATES

## Abstract

Hyperspectral imaging (HSI) is a promising technology for environmental monitoring with a lot of undeveloped potential due to the high dimensionality and complexity of the data. If temporal effects are studied, such as in a monitoring context, the analysis becomes more challenging as time is added to the dimensions of space (image coordinates) and wavelengths. We conducted a series of laboratory experiments to investigate the impact of different stressor exposure patterns on the spectrum of the cold water coral *Desmophyllum pertusum*. 65 coral samples were divided into 12 groups, each group being exposed to different types and levels of particles. Hyperspectral images of the coral samples were collected at four time points from prior to exposure to 6 weeks after exposure. To investigate the relationships between the corals’ spectral signatures and controlled experimental parameters, a new software tool for interactive visual exploration was developed and applied, the HypIX (Hyperspectral Image eXplorer) web tool. HypIX combines principles from exploratory data analysis, information visualization and machine learning-based dimension reduction. This combination enables users to select regions of interest (ROI) in all dimensions (2D space, time point and spectrum) for a flexible integrated inspection. We propose two HypIX workflows to find relationships in time series of hyperspectral datasets, namely morphology-based filtering workflow and embedded driven response analysis workflow. With these HypIX workflows three users identified different temporal and spatial patterns in the spectrum of corals exposed to different particle stressor conditions. Corals exposed to particles tended to have a larger change rate than control corals, which was evident as a shifted spectrum. The responses, however, were not uniform for coral samples undergoing the same exposure treatments, indicating individual tolerance levels. We also observed a good inter-observer agreement between the three HyPIX users, indicating that the proposed workflow can be applied to obtain reproducible HSI analysis results.

## Introduction

Hyperspectral imaging (HSI) originally used for remote sensing [1] has nowadays been established in multiple fields and applications, such as medicine [2], agriculture and forestry [3], food quality [4, 5], structure monitoring [6], material research [7], mining applications [8] and environmental monitoring [9, 10]. Organisms in particular exhibit a specific reflectance spectrum upon illumination, mainly caused by their pigment composition [11]. This is known as their spectral signature and can be studied using HSI. For non-invasive monitoring of environmental stress, HSI of selected organisms may have the potential to become one of the key tools. Most of the related studies have been carried out on photosynthetic organisms, especially on terrestrial vegetation and crop [12, 13] and shallow water coastal habitats. However, there is limited knowledge on the spectral responses of marine organisms in the deep sea to environmental or physiological stress.

Underwater hyperspectral imagers (UHI) for recording HSI have been implemented on underwater vehicles *in situ* for mapping extent and distribution of habitats [14, 15] such as deep-water corals and coralligenous habitats [16], red calcareous algae and associated fauna [17] or deep sea megafauna [18]. In recent years, few multivariate image analysis applications [19] have shown that objects of interest can be identified based on their spectral signatures [20, 21]. Further, UHI has been used to visualize the extent of particulate mud and drill cuttings on the seafloor [22] with HSI. The UHI system has been utilized to detect changes in the health condition of *Desmophyllum pertusum* (Linnaeus 1758) exposed to the hydrocarbon-2-Methylnaphthalene in laboratory experiments [21]. This was done in order to establish a basic understanding on how a potential subsea oil spill could affect the spectral properties of corals, and assess if hyperspectral imaging could be a valuable tool to detect such changes in the spectrum.

Although the HSI technology has been established in many fields and a small number of successful UHI applications have been reported, methodological challenges remain. Marine environmental monitoring in the field using UHI has mainly been constrained to identification and quantification of organisms and habitats, as well as the extent of particle sedimentation. There are no established methods for monitoring the physiological condition of deep-water marine organisms *in situ* using UHI. The rationale behind utilizing UHI for this purpose is that there must be a link between the health of the organism and its spectral signature. From nature, it is well known that coloration may be linked to health. For instance, for tropical corals, it is established knowledge that coral bleaching is a process triggered by the loss of the symbiotic photosynthetic algae component due to environmental unfavorable conditions, and this is clearly observable using spectroscopy. The beforementioned study (Letnes et al [21]) showed a link between spectrum and exposure level to hydrocarbon. Yet there is a limitation that there is an absence of established health metrics or indices that correlate physiological parameters with spectral response. Establishing such metrics requires *ex situ* experimentation with controlled stress exposures, and tools for interpreting resulting spectral images. A second limitation is the absence of an established analytical approach for assessing spectral changes over the same geographical area over time using UHI. Both of these two knowledge gaps are addressed by the works presented here.

Since there is no established methodological approach we propose a new software-based approach for the comparative analysis of such a large number of HSI data sets. The analyses of HSI data in general is not straightforward due to volume and dimensionality of the data, i.e. the huge number of wavelength acquired simultaneously, even for a small number of data sets recorded at one time point. The sheer dimensionality of the data typically makes an ad-hoc visual analysis with standard methods from exploratory data analysis unfeasible. In order to reduce the volume of data, it is often (spatially) filtered beforehand, by a heuristics-driven selection of e.g. a small number of regions of interest (ROI) in the lateral image domain (through selection of a polygon or a frame in the pixel grid). While this is a valuable established approach it limits the chances to find new relationships outside these ROIs. Another way to make HSI data easier to interpret, is the selective filtering of the high dimensional spectral domain to a relative small number of bandwidths (intervals), referred to as multispectral imaging [21, 23]. This is usually motivated by the observation that HSI data often feature low intrinsic dimensionality. The downside of this strategy is that it requires some domain knowledge about potentially interesting bandwidths a priori which is often not available. Sometimes dimension reduction techniques such as principal component analysis (PCA, [24]), multidimensional scaling, isometric mapping, or t-distributed stochastic neighbor embedding (t-SNE, [25]) are proposed [26, 27]. Due to the low intrinsic dimensionality, the spectral data can be well embedded in a two-dimensional data space, suitable for visualisation, which additionally reduces noise. A downside can be that the pixel-associated relation between the original spectral signal space and the embedding space can be lost if not covered by an appropriate software solution and that the much lower dimensional representation can be prone to misinterpretations due to the non-lossless embedding. Thus, a comparative analysis of the spectral signatures from different HSI data sets recorded at different time points must be supported by a flexible interactive data visualization tool, integrating the spatial, the spectral and the temporal domain. And since HSI data is not straightforward to interpret, an interdisciplinary approach to its analysis, integrating knowledge from disciplines, such as (bio-)chemistry, marine biology, statistics and computer science is required. As a consequence it is important, that the visualization tool features good usability and can be used online as a web-tool without technical requirements.

To address the problems described above, we present and analyse HSI data from controlled dose-response experiments on *D. pertusum* cold water corals (CWC) in laboratory tanks. The reef building coral *D. pertusum* has a wide geographical distribution and can be find in most part of the world, with the highest reported density in Norwegian waters. The reef offers habitat for a variety of species, and is described as a hot-spot for biodiversity. *D. pertusum* is defined as a ‘Near threatened’ species, mainly due to mechanical damages on the reefs caused by past and ongoing trawling. Further, there are concerns on the species ability to tolerate increasing ocean temperatures and ocean acidification [28]. *D. pertusum* is also located in areas with petroleum activities and several studies have therefore been performed to monitor potential impact of and to establish threshold for drilling particulates to these corals [29–33]. The objective of this study was to investigate the future potential of non-invasive HSI technology for detection of changes in the health status by exploring potential systematic changes in the spectral composition with various exposure concentrations of drilling particulates of adult CWC *D. pertusum* as a response to certain exposure levels of suspended drilling particulates. Drill particulates are a collective expression for drill cuttings (formation rock particulates generated during drilling) and weighting agents added to the drilling fluids, such as barite and bentonite (use to lubricate and control the well during drilling) [34]. To this end coral samples of both the white and the orange *D. pertusum* phenotype were exposed to drill cuttings, bentonite and barite in a laboratory setting, mimicking a realistic offshore drilling scenario. After exposure, a small HSI time series was recorded for the CWC samples.

Following these considerations we developed a new tool for HSI analysis, referred to as HyPIX, presented in this paper. With this tool we investigated the HSI data collected in our experiments with the main objective to find response patterns in the coral spectra related to exposure type, levels and/or time points in the corals. A second objective was to find a HyPIX—based workflow to spatially locate such responses in the sample (in case these changes are not observed for all sample pixels) and to describe observations in the HSI that are reproducible.

## Materials and methods

**Fig 1:**
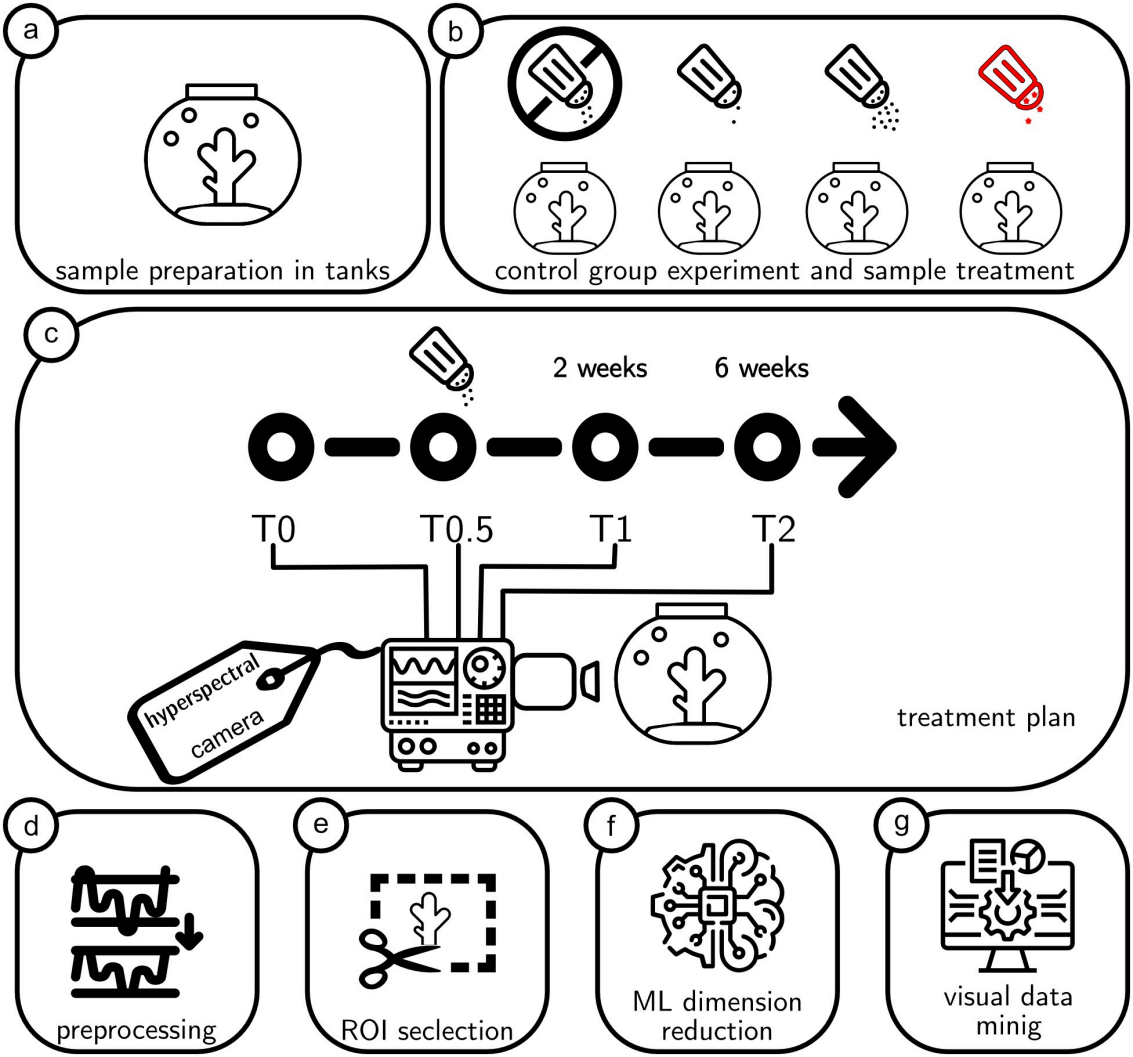
Overview of the Hypix workflow. a) Corals are kept in glass aquariums. b) Different aquariums are exposed with either barite, bentonite, drill cutting or kept as is for the control experiment. c) The exposure happens at a timepoint T0.5. Before exposure (T0), directly after exposure (T0.5) and after 2 (T1) and 6 weeks (T2) of recovery hyperspectral images are taken. d) The images are preprocessed. e) The outline of the corals is annotated in Biigle 2.0 and the individual corals are cut out for further processing. f) Data mining and machine learning methods are applied and saved to a file for g) Visualization and analysis of the results in the Hypix system. For more details on each step please have a look at the respective subsection in the Method section.

An overview of the Hypix workflow is shown in Fig 1. For details consult the following text.

### Experimental setup

Samples of coral colonies were collected from Trondheimsfjorden, Trondheim, Norway April 2018 using remotely operated vehicle (ROV). The sampling was carried out outside national parks or other types of protected areas. The corals were transported to NORCE marine facility, Mekjarvik, Randaberg where they were acclimatized for three weeks according to Baussant et al. 2017 [35]. The sampling of corals for current study was done in conjunction with sampling for study reported by Baussant et al. 2022 [29], where further details on sampling are to be found.

The corals were contained in tanks with running seawater from Byfjord (Rogaland, Norway) in stable temperature conditions (7.5°C) and dark surroundings. They were fed with Artemia larvae approximately two times per week. Prior to the experiment, the sampling material was distributed to smaller coral nubbins, each nubbin with approximately 5–10 polyps. Both white and orange morphs were used.

All exposure experiments (controls, barite (bar), bentonite (ben) and drill cutting (DC)) together with the HSI measurements were conducted from June 2018 to February 2019. For each experiment a separate glass aquarium (50cm × 30cm × 30cm) with a flow rate of 180±10*mL* ⋅ min^−1^ sea water was used. In each, 5 (bar/ben) or 6 (DC) coral nubbins of both color morphotypes (white and orange) were placed. Each nubbin was approx. 5–15cm in size and had 5–10 polyps. The corals needed to be visible for two camera observations (a digital consumer time-lapse camera and the hyperspectral imaging), and were therefore placed on a diagonal line (approx. 30°, S1 Fig in the supplementary). The bottom and backside of each aquarium was covered with black insulating material to facilitate HSI and time-lapse imagery analysis.

#### DC exposure

The exposure to suspended DC particles on the corals was made as reported in [29]. DC was added in pulses of 4 hours followed by 4 hours with no DC over a total duration of 5 days. A peristaltic pump added 2*mL*. DC stock min^−1^ from two 30L stock tanks to the experimental aquariums, where DC mixed with the waterflow supplied at 200±30*mL* ⋅ min^−1^ to achieve peak exposure nominal concentrations of 10, 30, 50 and 100*mg* ⋅ L^−1^ (please see Fig 1 of [29] for more details). Actual peak concentrations were measured from point seawater samples (250*mL* to 1*L*, depending on expected concentration) collected each day from the aquariums during the 4-hour exposure cycle 1 hour after DC pump start to insure steady-state equilibrium was reached. Samples were filtered over a GF/F Whatman filter, and the filter was dried (60°*C*) overnight or until constant weight to obtain the total particle weight from which the DC concentrations were derived.

There was a deviation from the nominal to the actual measured DC concentrations by water filtration. The mean actual DC concentrations were 4, 6, 18 and 41*mg* ⋅ L^−1^, respectively corresponding to DC nominal concentrations of 10, 30, 50 and 100*mg* ⋅ *L*^−1^.

#### Barite and bentonite exposure

Barite and bentonite exposure experiments were carried out with the same experimental setup and exposure scenario as for DC, but the 10*mg* ⋅ L^−1^ concentration was not tested. The actual concentrations measured in the different barite/bentonite treatments were also lower than the target concentrations: For barite, actual measured concentrations were 5.8, 18 and 54.7*mg* ⋅ L^−1^ (corresponding to respectively 30, 50 and 100*mg* ⋅ L^−1^) and for bentonite, this was 9.9, 17.1 and 44.1*mg* ⋅ L^−1^ (corresponding to respectively 30, 50 and 100*mg* ⋅ L^−1^).

#### Hyperspectral imaging

The underwater hyperspectral imager (UHI) applied in our experiments for recording HSI, is a push-broom type underwater hyperspectral sensor with a narrow slit, spectrograph, hyperspectral line camera placed in a waterproof housing. Since it is a push-broom imager, either the object or camera needs to move, to enable imaging of a scene. This was implemented by using a platform moving the camera along the vertical axis (S2 Fig in the supplementary), capturing hyperspectral images perpendicular to the direction of movement within the wavelength range 380 − 750*nm* (Setup modified from [21]). Recording was controlled through the data acquisition software Immersion on a top -side computer, connected to the sensor through a sub-sea ethernet cable. During image acquisition the scenery was illuminated with halogen lamps from above (Osram Decostar 51 TITAN 50 W 12 V 60° GU5.3) with constant power supply. Radiometric correction was carried out on the raw hyperspectral image in order to correct for dark current and sensor specific noise, resulting in a radiance image. However, for comparability with other studies reflectance was estimated for a selection of pixels (ROIs) from all exposure treatments (see S1 Text and S3 Fig).

#### HSI data

A time series HSI dataset was obtained for all samples at four selected time points throughout the experiment: Prior to exposure (T0), directly after the exposure (T0.5) and two weeks (T1) and six weeks (T2) after the exposure, into the recovery period. As the HSI applied in our setup described above recorded one large hyperspectral image of all corals for each time point *t* ∈ {T0, T0.5, T1, T2} including a lot of pixels with background a first step was to split the large HSI showing five (barite/bentonite exporsure) or six corals (DC exposure) into single HSI (each one showing one sample). Afterwards the individual coral samples were outlined and annotated in the single HSI using BIIGLE 2.0 [36] annotation software. Each single data set is referred to with $H_{t,c}^{n}$, with *c* denoting contaminant and concentration level, *t*: time point (T0, T0.5, T1, T2) and *n*: sample ID (0, …, 4 or 5). $H_{c}^{n}$ refers to the time series of all HSI recorded for sample *n* in exposure experiment *c* and *H*_*t*,*c*_ refers to all HSI recorded at one time point with the same exposure concentration. The term $H_{t,c,x,y}^{n}$ refers to the spectrum at position (*x*, *y*) in the measurement of sample *n* at time point *t* and contamination *c*. The intensity value for a given wavelength *s* at one position is given by $H_{t,c,x,y,s}^{n}$.

### Analysis of HSI data: HypIX

**Fig 2:**
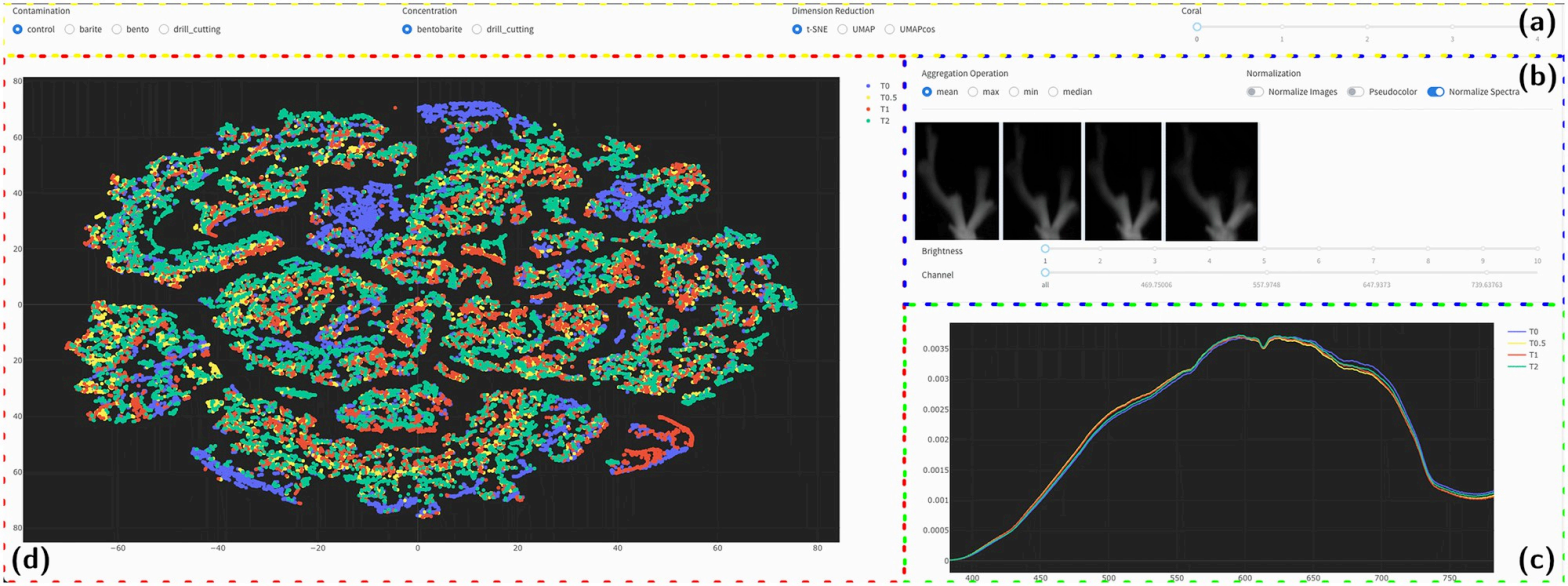
Screenshot of the HypIX tool: In the top frame (a) the experimental conditions, concentration levels, dimension reduction algorithms and individual coral samples (0, …, 4 or 5) can be chosen by the user. On the right (b) a pseudo image of each HSI of all four time steps is shown in the **image display**, in this case the mean spectral response value (see Methods for details). On the right side in frame (b) the user can chose to apply image normalization or use pseudocolor to change the images visualization. In frame (c), spectral signatures from all four time steps are shown in the **spectra display**. This window initially shows the agglomerated spectral signatures from all four data sets color encoded (T0: blue, T0.5: yellow, T1: red, T2: green. (d) The large frame on the left (d) shows the dimension reduction results for the four HSI data sets in the **embedding display** using again the same color code for the four time points.

In order to analyse the HSI data, i.e. to find relationships between changes in spectral signature through time and the exposure levels applied, we present the new HSI exploration webtool HypIX (Hyperspectral Image eXplorer) that was implemented and employed in this work. HypIX and the data sets used in this study are available online at https://webserver.biodtmin.projects.bi.denbi.de/hypix using the username *coral* and the password *hypercoral2020*. An example display of the HyPIX interface is shown in Fig 2 for a first impression and overview. After starting HyPIX, a data set (i.e. experimental run and sample) and other methodological parameters are selected (see Fig 2a). Afterwards the data set can be explored and filtered in three different domains simultaneously, namely the spatial (or lateral) image domain (cmp. Fig 2b), the spectral domain (cmp. Fig 2c) and the data embedding domain (cmp. Fig 2d). Methodological details behind the HyPIX modules, data pre-processing and the two HyPIX workflows applied in our study are described in the following. In the formal description of the processing steps we will from now on omit the experiment index *c* and time point index *t* as all HSI were treated the same. Instead we will use spatial coordinates (*x*, *y*) and the spectral wavelength *s* to describe the processing steps.

#### Spectral domain visualization

The spectra display (Fig 2(c)) shows the four spectra of the four HSI (recorded at *T*_0_, *T*_0.5_, *T*_1_, *T*_2_) for one sample *n*. The spectra $H_{x,y}^{n}$ are visualized either for all pixels or just for a subset of pixels selected in the image display (Fig 2(b)) if particular morphological / structural elements are of interest. If a selection is made containing more than a single spectrum an agglomeration spectrum ${\hat{H}}_{s,\cdot }^{n}$ is shown. If no selection is made at all, an agglomeration spectrum of all spectra per sample *n* is shown. Different agglomeration options can be used. When using arithmetic mean as agglomeration option it yields the mean spectral signal intensity per sample

$$\begin{array}{c} {\hat{H}}_{s,\text{mean}}^{n}=\frac{\sum_{x,y}H_{x,y,s}^{n}}{w*h}, \end{array}$$

Analogously, instead of the arithmetic mean operation, the max(), min() or medium() operation can be used for the agglomeration step, i.e. the maximum spectral intensity ${\hat{H}}_{s,\text{max}}^{n}={\text{max}}_{x,y}\left(H_{x,y,s}^{n}\right)$, minimum spectral intensity as ${\hat{H}}_{s,\text{median}}^{n}=\min_{x,y}\left(H_{x,y,s}^{n}\right)$ or median spectral intensity respectively ${\hat{H}}_{s,\text{median}}^{n}=\min_{x,y}\left(H_{x,y,s}^{n}\right)$ can be chosen in the HyPIX interface (see upper left in the frame Fig 2(b)).

Additionally, an optional normalization step can be applied to the spectra using the *l*_1_-norm, i.e. for each spectrum $H_{x,y,s}^{n}$ we create a normalized spectrum ${\tilde{H}}_{x,y,s}^{n}$ with

$$\begin{array}{c} {\tilde{H}}_{x,y,s}^{n}=\frac{H_{x,y,s}^{n}}{\sum_{s^{\prime }}\left|H_{x,y,s^{\prime }}^{n}\right|}. \end{array}$$

#### Image domain visualization

To visualize the data in the image domain, so users can assess the morphology, we compute different representative pseudo grey-value images $I_{t,c}^{n}$ for each HSI $H_{t,c}^{n}$. In the following we will consider the case of one HSI and omit the indices *t*, *c* for the sake of compact writing. Users can select the mean spectral image, i.e.

$$\begin{array}{c} I_{x,y}^{n,\text{mean}}=\frac{\sum_{s}H_{x,y,s}^{n}}{S}\sum_{s}\underset{s}{\text{max}}\left\{H_{x,y,s}^{n}\right\} \end{array}$$

or the maximum spectral image $I_{x,y}^{n,\text{max}}={\text{max}}_{s}\left\{H_{x,y,s}^{n}\right\}$ or the minimum spectral image $I_{x,y}^{n,\text{min}}=\min_{s}\left\{H_{x,y,s}^{n}\right\}$, or the median spectral image, i.e. $I_{x,y}^{n,\text{median}}=\text{median}\left\{H_{x,y,s}^{n}\right\}$.

In addition we provide the option to normalize the images, i.e. to linearly scale the values to [0, 255] and a pseudocolor visualization option for the pseudo gray-valued image $I_{x,y,s}^{n}$. In this case, the gray scale pseudo images are mapped to colors with a lookup table using the matplotlib [37] spectral colormap. Further user options offered in HypIX are to increase the brightness *b* of the images by multiplying *b* with the pseudogray/-color image, i.e. $b*I_{x,y,s}^{n}$. Instead of showing an agglomerated pseudo grey-value image an image for a certain spectral channel $\hat{s}$ can be shown, i.e. $I_{x,y,\hat{s}}^{n}$.

#### Embedding domain visualization

To visualize the data from an HSI time series in one scatter plot (see embedding display in Fig 2(d)) each HSI time series is mapped to a two-dimensional space using dimensionality reduction. Thus, $H_{x,y,s}^{n}\in R^{w\times h\times S}$ is transformed to $D_{x,y,d}^{n}\in R^{w\times h\times 2}$, referred to as the embedding domain. As the computation of the two-dimensional projection is time-consuming it cannot be done in real-time, so the embeddings must be computed beforehand, saved and then loaded into HypIX on demand. Because of this the normalization option mentioned above is used mandatorily. For dimensionality reduction we use two different state-of-the-art methods namely *t*-distributed stochastic neighbor embedding (t-SNE [25]) and Uniform Manifold Approximation and Projection (UMAP [38]). For the UMAP method we have tested two different metrics, the euclidean metric and the cosine angular metric. The cosine angular metric in contrast to the euclidean metric is independent of the length of the vector and thus in our case neglects the amplitude of the spectra, thus looking only at the distribution of the spectra.

#### HypIX interface and functions

After selecting the HSI data from one time series experiment (i.e. exposure level or control), one sample (see frame (a) in Fig 2), HypIX displays pseudo gray-valued images of the four HSI data sets in the **image display** (see (b) in Fig 2), an agglomerated spectrum for each HSI data set in the **spectra display** (see (c) in Fig 2) and a dimension reduction scatter plot that was computed for all spectra from the four HSI data sets in the **embedding display** (see (d) in Fig 2). In the embedding display or in the spectral display the data from different time points can be selected and de-selected in order to focus on the comparison of for instance only two measurements.

The four displays are functionally linked to allow interactive dynamic selection, filtering and highlighting of data subsets in one display with a simultaneous highlighting of the same data in the other displays, which is also referred to as gating or *link and brush* in the information visualization community [39] (cmp. Fig 2a–2c). The (de-)selection of data groups in one display is propagated to the other different displays, i.e. if a subset of data is selected in the embedding display (cmp. Fig 2a)) the location of this selection is highlighted as a ROI in the image domain (Fig 2b)) and the mean spectra are depicted in the spectrum display (Fig 2c)). The selection of single data points in the embedding display furthermore allows the inspection of the spectrum of a single hyperspectral pixel (Fig 2d)).

In addition to this functionality, the HypIX tool also offers the possibility to show a PCA biplot of the data chosen in the embedding display. The PCA biplot shows a PCA dimensionality reduction to the two-dimensional plane as well as a visualization of the PCA loadings. The dots correspond to individual coral pixels, while arrows correspond to the variable loadings of individual wavelength variables (pseudo-colored according to the color they represent). Although PCA is possibly less powerful than, e.g., UMAP when it comes do differentiating between spectral samples in a two-dimensional data space, it arguably represents a means of dimensionality reduction that is easier to interpret. By inspecting a biplot, it is for instance possible to coarsely relate observed spectral differences to the wavelengths causing them. This is a useful property that potentially may guide more targeted analyses performed subsequently. For a more in-depth discussion on PCA biplots please have a look at the respective literature [40].

All these HypIX functions can be used to conduct several different approaches to data exploration. This allows an analysis of the data in three domains simultaneously and to develop new workflows to guide users in the analysis of HSI data.

HypIX is built using the Dash library and the Python programming language. For data processing we use numpy [41], scikit-learn [42], umap-learn [38] and h5py [43].

#### Morphology-based filtering workflow

This workflow is motivated by the standard approach of first selecting ROIs in the spatial domain and investigating the average spectra from these ROIs and their differences or similarities through the time series. In HypIX, the ROIs can be selected in the image display by drawing polygons or rectangles. Since the HSI shown in this study are not spatially aligned (or registered), users need to be very careful to select ROIs in each of the four pseudo-color images that show corresponding morphological substructures (like calice) in all four images. After the ROI selection, the other two displays are updated and the corresponding points are highlighted in the embedding display and the spectra display now shows the agglomerated spectra from the four ROIs.

#### Embedding driven response analysis workflow

**Fig 3:**
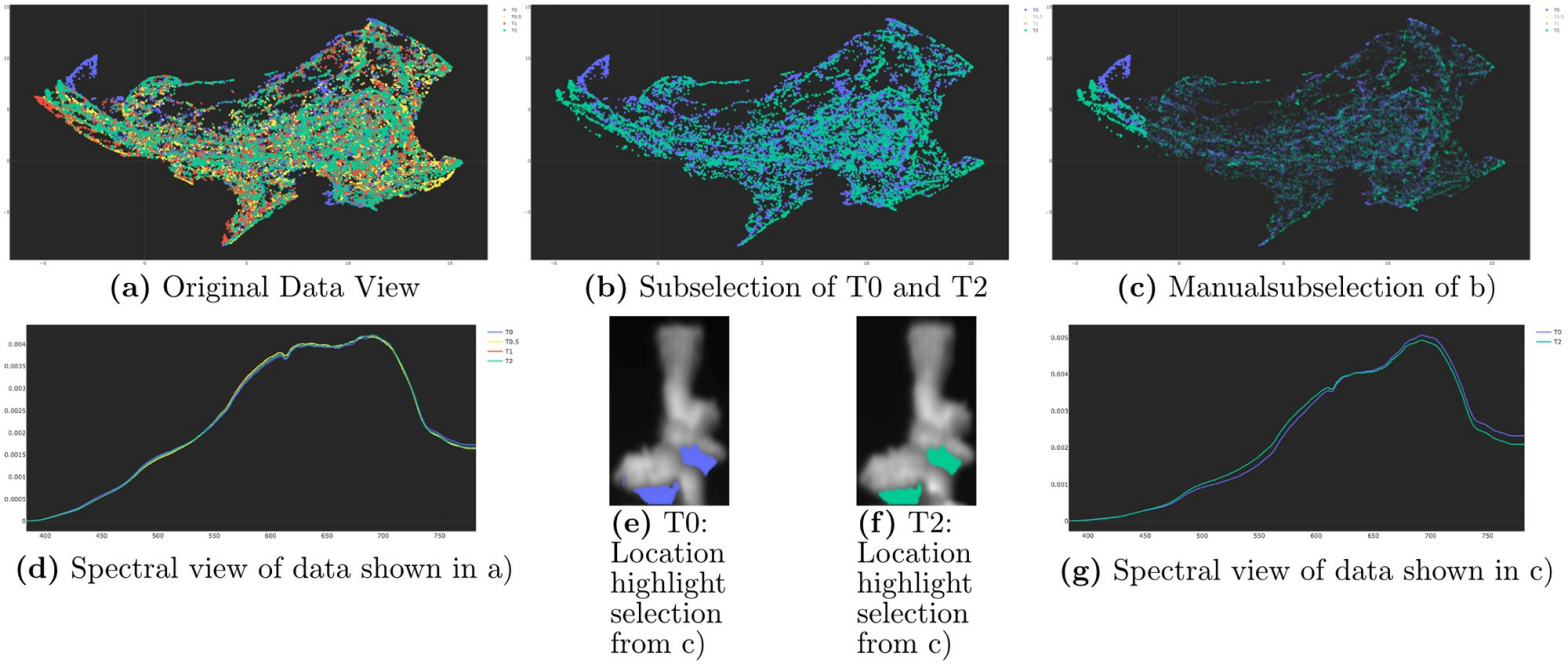
Local changes revealed with the interactive subselection of the hyperspectral viewer, otherwise shadowed by global analysis. Shown is the bentonite sample with 100*mg* ⋅ L^−1^ concentration using a UMAP with cosine metric and coral 3. The normalization of spectra was activated and the aggregation option was set to mean.

We introduce the concept of embedding-driven ROIs in contrast to anatomy/morphology driven ROIs, as described above. The idea is to detect areas of spectral shifts and changes not based on a spatial hypothesis but based on patterns in the spectral data points from two or more time points. One example for results obtained with such a workflow is shown in Fig 3 and explained in detail in the Results section. First, a group of points is selected in the embedding display using the mouse and a lasso or rectangle selection tool. Sometimes it is beneficial to limit the embedding display to data from two time points only (e.g. comparing the spectral data recorded at T0 to that recorded at T2). The average spectra from these selections are presented in the spectra display and the locations of these spectra are highlighted in the image display. Using this workflow, regions in the embedding display that are populated by data from only one time point can be selected. This causes the image display to highlight these points. Now we can select the same regions for the other time points in the image display. This in turn causes the embedding display and the spectra display to be updated, showing local spectral differences over time.

#### HyPIX application experiment

**Fig 4:**
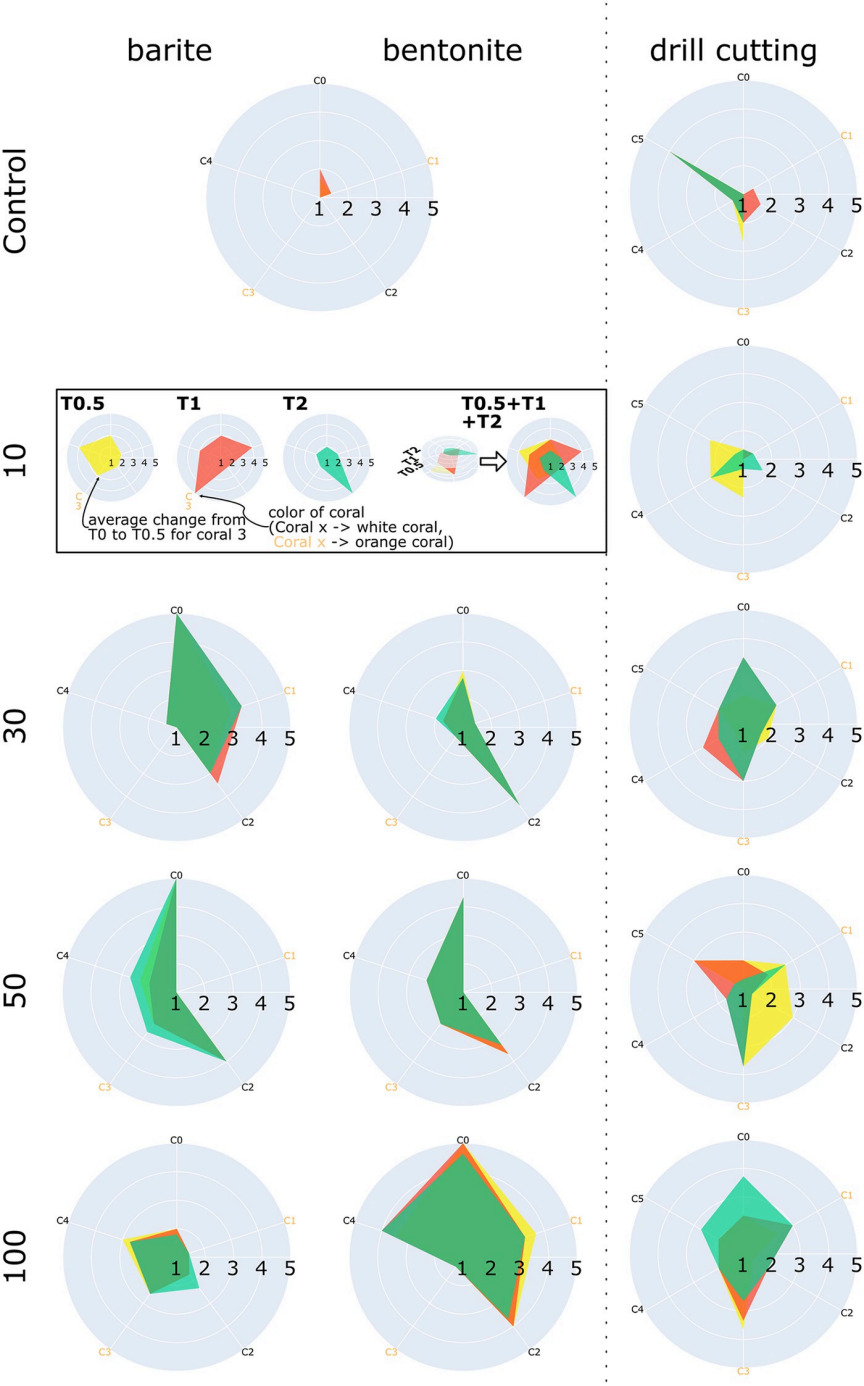
Subjective rating results of the changes from initial time point T0 to final time point T2 of the bentonite, barite and DC experiment after six weeks. All values represent the average over all subjective ratings. Ratings range from 1 (no change) to 5 (strong change). Analogues to the color scale of the Hyperspectral Viewer, yellow is T0.5 (directly after exposure), red is T1 (two weeks after exposure) and green is T2 (six weeks after exposure). The titles of the subgraphs are the concentrations in mg ⋅ L^−1^ of the exposure depicted as the row title.

Three users (one bioinformatician, two marine biologists), independently applied HypIX and the workflows described above to investigate the collected HSI times series. Each time series was rated regarding the changes observed between the initial time point T0 to the other time points T0.5 (right after exposure), T1 (after two weeks) and T2 (after six weeks). Since many ROIs described only small parts of the samples, these changes cannot be found using a holistic approach, e.g. by comparing average spectra computed for entire samples. Therefore, a tool such a HypIX is required to find these changes. Each user independently rated each HSI data $H_{t,c}^{n}$ with *t* ≠ T0 regarding the degree of change in comparison with its initial pre-exposure state HSI $H_{T0,c}^{n}$. All changes appeared only in specific parts of the corals, so the ROI $H_{t\neq \text{T0},c}^{n}$ describing this part showed a spectral signature considerably different to the signature from the same ROI in the T0 measurement. The users’ rating decision was noted as $r\left(H_{c}^{n}\right)\in \left\{1\left(\text{no}\;\text{change}\right),2,3\left(\text{average}\;\text{change}\;\text{level}\right),4,5\text{(highest}\;\text{change}\;\text{level)}\right\}$ and the detailed rating result is given in S1 and S2 Tables in the supplementary. After all HSI have been evaluated by the users for each sample’s time series $H_{c}^{n}$ the average change rating ${\hat{r}}_{c}^{n}\in \left[1,\ldots ,5\right]$ was computed from the three users’ ratings. In Fig 4 the average change rates for the control group experiments and each exposure level experiment for bentonite, barite and DC exposure are summarized as one star glyph per experimental condition, i.e. exposure concentration (see graphical explanation of the star glyph in the box in Fig 4).

## Results

The first observation in Fig 4 was that the users did not find many changes in the control group but identified stronger changes in the exposure experiments. Although, the corals did not all react with the same intensity for one exposure level, the trend that there were at least two corals reacting stronger to the exposure is visualized by a larger area in Fig 4. In the control group of the DC experiments some users noted a change in the embedding display for one coral sample (see “Coral 5” in the upper right starplot in Fig 4). A re-investigation of this case showed that in this case, the shift in the embedding space was caused by a mistake in the description of coral mask in the T0 data set, which was not noted by some users. In T0, the coral mask was too large and included non-coral pixels from the ground. As this was only the case in one time point, this resulted in two shifted groups of points outside the main point cloud in the embedding display. We decided not to repeat the experiment with a corrected mask in order to follow the planned experimental set up as strictly as possible so the workflow could be evaluated rigorously. Instead, in the supplementary S4 Fig we show the embedding results with and without the wrong part of the mask.

The second observation in the bentonite/barite experiment was that not all five corals in one exposure experiment showed a reaction to the exposure in the spectral signatures. Just a subset of three to four corals showed an average change rate $r(H_{c}^{n})>2.5$. This kind of individual response behavior was an interesting observation as it motivates the investigation of single samples instead of analysing all spectra from all samples together, which is usually the case.

The third observation was that the exposure concentration did not seem to impact the degree of change in the barite exposure experiments but it seemed to play a role in the bentonite experiments.

In addition we observed a trend for the reaction speed of the corals for bentonite and barite on the one hand and DC on the other hand. Many corals treated with bentonite and barite showed an immediate reaction to the exposure at time point T0.5 but no further change at the following time points. The corals exposed to DC solution reacted at T0.5 and then seemed to go back to a state with lesser change when compared to T0.

In addition to these general observations based on the subjective ratings $r(H_{c}^{n})$ we found more specific patterns through the interactive nature of HypIX, i.e. the link and brush between embedding, image and spectra displays. In Fig 3 we showed an example where the subselection of data in the embedding display and linkage between different displays led to the identification of a local change. In the initial state (no filtering applied and all data from T0, …, T2 is shown) the embedding and the spectral displays showed only small differences in point distributions and the spectral composition looked exactly the same (cmp. Fig 3(a) and 3(d)). Hiding data from two time points, i.e. T0.5 and T1 (cmp. Fig 3(b)) hinted to a ROI in the embedding display (see Fig 3b upper left), where the data distributions differed between T0 (blue) and T2 (green). In a subsequent step we selected this suspicious area in the data display (cmp. Fig 3(c)), which triggered the link and brush of the location view (cmp. Fig 3(e) and 3(f)), as well as of the spectral view (cmp. Fig 3(g)). From the Fig 3(e) and 3(f) we saw that the data distribution represented indeed the same location, however, the spectral composition (cmp. Fig 3(g)) differed significantly from T0 to T2. The curves cross at approx. 630 nm, suggesting a change in spectral properties towards a less red and more flattened spectrum. This example showed that these changes cannot be analysed with a technique using a single modality. When we only looked at the unfiltered spectra (cmp. Fig 3(d)) we could not point out any difference, because it was shadowed by the mean of spectra. When we looked at the original data view (cmp. Fig 3(a)), we could probably see a change in data distribution, although this was already quite hard to see, because of the huge amount of data. Even if we could observe the change in data distribution, we could only state that there might be a change and there is still the slight possibility of a misleading embedding of the data causing this change in distribution. What this change looked like in the spectral domain or where it was located is hidden from a mono-modal analysis. In addition using t-SNE or UMAP with euclidean metric this change in data distribution could not be spotted as clearly as with the UMAP using a cosine metric. In contrast our results showed the potential of the multi-modal HypIX functions in HSI analysis.

**Fig 5:**
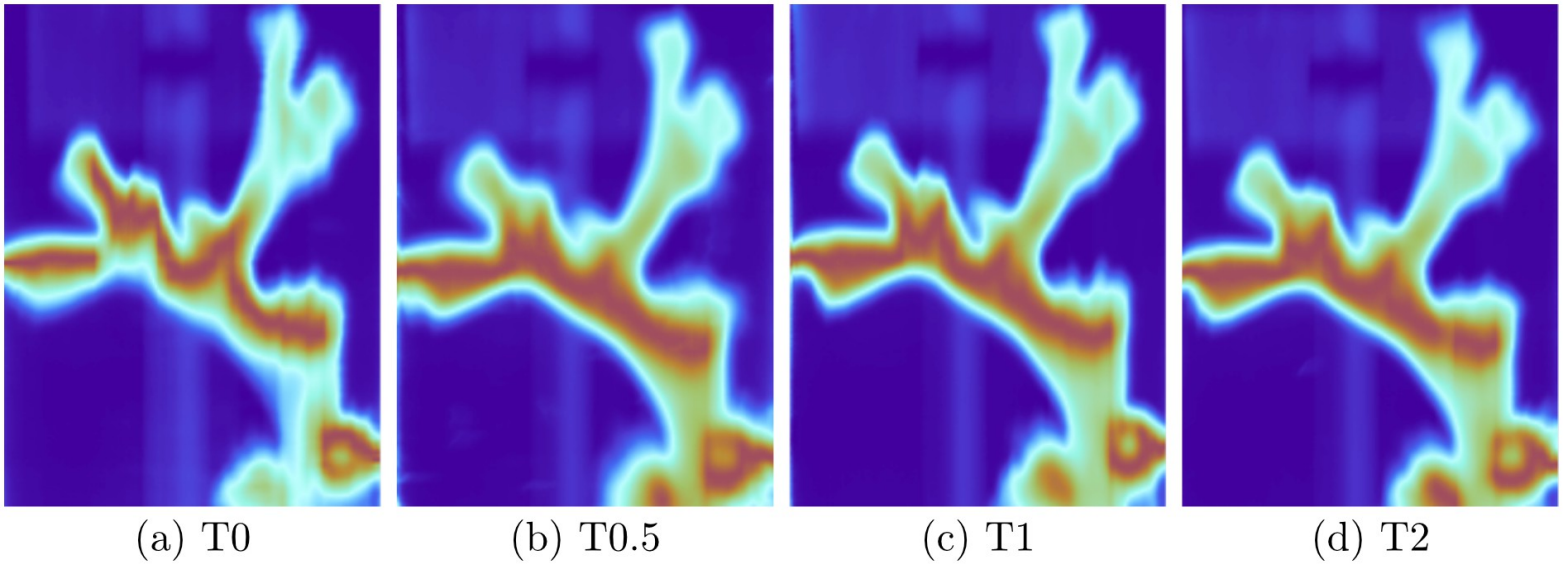
Pseudocolor images of each timepoint. We can clearly spot differences between time point T0 and the other time points. a) start of experiment (T0); b) immediately after exposure (T0.5); c) after 2 weeks of exposure (T1); d) after 6 weeks of exposure (T2).

Another option to quickly generate a hypothesis about changes from time point to time point was the pseudocolor option (cmp. Fig 5). A change in color meant that there was likely a change in the spectral composition, which could be validated using the spectral and the data view in successive steps. Furthermore a single channel could be selected to be presented as a pseudocolor image to analyse changes in a specific spectral band of interest.

## Discussion

Hypix was evaluated by a focus group of users and they liked the look and feel. Problems that arose were fixed in the final version of Hypix. Users particularly appreciated the link and brush functionality, i.e., interactive filtering of data in one modality that links changes in the other modalities. This opened new ways of exploratory data analysis in hyperspectral imaging, which can lead to the generation of future research hypotheses.

The two proposed workflows led to results that were mostly consistent between the users (standard deviation 0.39, also cmp. S5 Fig in the supplementary). The low degree of variation indicates that the software and the proposed workflow can be applied for HSI analysis to achieve results that are reproducible.

The fact that the method is vulnerable to mistakes in the definition of the mask (cmp. “Coral 5” in the Control experiment of the DC exposure in Fig 4) may be considered acceptable as any kind of analysis that uses a mask description would potentially suffer from such kinds of error. One may even consider it a strength of this interactive visualization-based approach that this imprecise mask definition was detected in the experiment.

**Fig 6:**
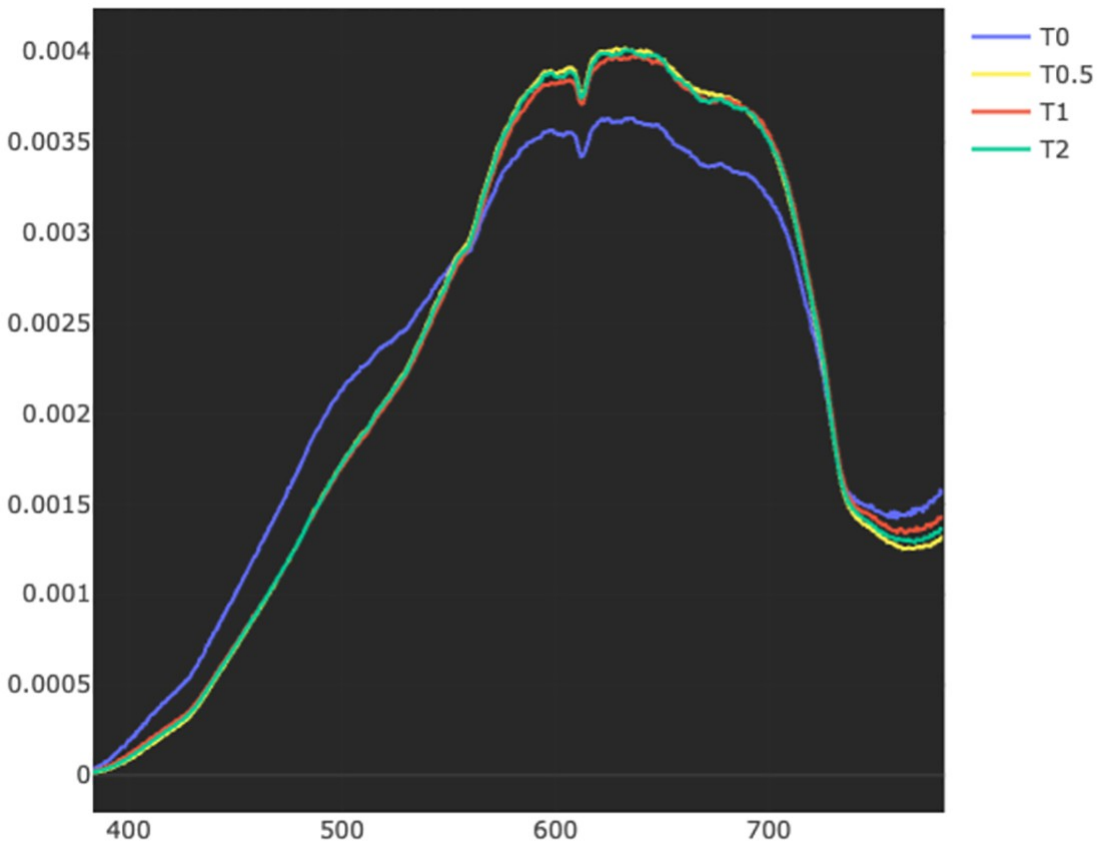
Screenshot of the spectral view of Hypix. Example for a red-shifted spectrum due to the exposure with a barite concentration of 30*mg* ⋅ L^−1^ for coral 0. The blue part of the light spectrum is lowered while the red part is elevated at T0.5, T1 and T2 compared to before the exposure at T0.

A clear defined exposure-related spectral change was not ubiquitously observed for all the coral samples in any of the treatments. It is therefore difficult to model a relationship with respect to specific exposure levels and their general impact on coral color based on our experimental output. A notable pattern, however, was that exposure to bentonite and barite had a higher effect on the spectral properties of white corals than on orange ones (cmp. Fig 4). This is particularly interesting considering that findings from a recent study by Büscher et al. [44] indicate that the white *D. pertusum* phenotype may be less stress-resistant than the orange phenotype. For the white corals where a spectral change was observed, the change was typically manifested as a red-shifted spectrum (i.e., lowered blue values and elevated red values, cmp. Fig 6). The ultimate cause of this spectral shift is yet to be determined, and consequently a topic that warrants further investigation.

Provided that our observed spectral shift is a response to the exposure treatment, the non-uniform response of coral samples undergoing the exact same treatment indicates that individual corals may have individual tolerance levels. Further studies should seek to identify the reason for these differences in tolerance. One can speculate if factors such as age, reproduction, feeding availability and integrity of the coenosarc layer contributes to the fitness of the individual polyp.

In a recent tank study [29], *D. pertusum* was also exposed to various concentrations of particles associated with drilling operations. Notably, the study reported a significantly increased ratio of organic carbon to organic nitrogen (OC:ON ratio) in the mucus of corals exposed to bentonite concentrations >20*mgL*^−1^. Furthermore, a study on the effects of drilling particles on *D. pertusum* larvae found that bentonite affected larval behavior at lower concentrations than both barite and DCs [31]. This was attributed to the bentonite particles being finer, making them stick to the coral mucus more easily. The findings from both the aforementioned studies are interesting, as the greatest spectral changes in the current study also were observed for corals exposed to bentonite (see results obtained for the Bentonite 100 experiments, illustrated in Fig 4). A possible explanation for these observations is that the finer bentonite particles interfered with the coral mucus to a greater extent than both barite and DCs, and that this manifested itself as a detectable spectral shift. However, as the trend was not ubiquitous among coral samples exposed to high bentonite concentrations, this interpretation should currently be treated with caution. In the future, we recommend conducting particle exposure studies of *D. pertusum* where hyperspectral signatures and coral mucus properties are measured simultaneously. This could provide further insight into *D. pertusum*’s response to drilling operations and possibly help substantiate the observations related to bentonite exposure made in the current study.

In general, the shifts observed in the HSI of the corals can be described as colour shifts towards a more reddish colour. However, the shifts are too small to be recognized in the RGB image by the human eye. Colour shifts in *D. pertusum* corals have been reported before by Osterloff et al. [45], however on a much longer time scale (from May to September 2015) and with another possible explanation, i.e. a seasonal difference in the food supply (copepods). In the RGB images in this study of 2019, the colour shift was strong enough to be perceived comparing images recorded at time points four months apart. The underlying mechanisms of color changes in this species are not well known and only a small number of studies have been published on the topic so far. Letnes et al. [21] found a correlation between hydrocarbon exposure, coral mortality, and coral color change. Elde et al. [11] found different concentrations of pigments in the morphotypes. A color morph specific bacterial assemblage is reported by Neulinger et al. [46].

Besides the effects observed in the HSI data mentioned above, the rationale behind the application of hyperspectral imaging to assess the health status of cold water corals, is that I) the spectral properties of corals have been observed to change with declining health, and that II) these changes are observable with underwater hyperspectral imaging. In the following we will address these two points to provide more context for our findings.

Point I) is supported by the work of Letnes et al. [21] who showed a correlation between coral mortality and colour of the species. The corals undergoing mortality exhibited a loss of absorption properties at specific wavelength (560nm), which suggests a loss of pigment function. However the link between colour and levels of non-lethal health change is not well studied. Hyperspectral assessment from air is much used to assess the health of shallow water tropical corals. These corals form a symbiotic relationship with dinoflagellates, and with the loss of this dinoflagellate due to increasing water temperatures, the process of coral bleaching occurs. The process is reversible to a certain point, however if the symbiosis is not restored the coral will eventually die and the remaining carbonate skeleton will typically be inhabited by macro algae. HSI is used to monitor the extent of live corals, bleached corals and macro algae covered corals [47–49]. This is a related yet different scenario than health monitoring of the azooxanthellate *D. pertusum*. We have a fairly good overview of the typical live spectrum and the dead spectrum of the species [11, 21, 23, 50, 51], however, there is limited knowledge on spectral properties of the intermediate health, which would be equivalent to the bleached, but not dead tropical coral. Establishing further knowledge on the spectral responses is crucial if HSI is to give an early warning of changes in environmental conditions.

Point II) is motivated by the observation that knowledge on how coral spectral properties change with a declining health is rather limited. Thus, the discussion on the capability of HSI to detect these changes, should be taken with precaution. However, the fact that HyPIX can be applied to produce reproducible results obtained from visualizations can be interpreted as a new step towards increasing the significance of HSI data by improving the HSI data interpretation. Hence, we propose that HSI does have a potential to function as a tool for monitoring changing physiological conditions of marine organisms. Future studies should seek to understand the actual mechanisms involved in color change of corals. Emphasis should also be put on bringing the methodology from lab to field. An HSI field tool for detection of health changes in deep-water marine organisms could be utilized for monitoring of effects of offshore drilling operations and is also likely to be be valuable for other industries, for instance for monitoring of environmental footprints from sea based aquaculture production, where there are concerns regarding the the effects of organic wastes on filtering benthic fauna.

We show that this software-driven workflow shows great potential in the analysis of hyperspectral imagery. Although in this case the Hypix tool was tailored for this use case the method can be applied to other hyperspectral or multispectral data as well. The two workflows proposed in this research led to a streamlining of methods thus to reproducible results. We think that therefore not only the software is a valuable asset but also the workflows and methodology in general.

## Supporting information

S1 Table Individual subjective rating results for Bentonite and Barite experiments. (PDF)

S2 Table Individual subjective rating results for drill cutting experiments. (PDF)

S1 Text Extraction of spectral signatures for reflectance estimation. (PDF)

S1 Fig Laboratory set-up: Aquarium setup. Left: Photo of corals (white and orange) and polyethylene reference plate inside aquarium. Right: Sketch showing position of time lapse camera and underwater hyperspectral imager (UHI) outside the aquarium with coral nubbins.
(PDF)

S2 Fig Laboratory set-up: Setup for acquisition of hyperspectral images Coral samples and a polyethylene reference plate were placed at the bottom of aquarium. The hyperspectral imager was placed on a scanning rig outside the aquarium, scanning through the glass.
(PDF)

S3 Fig Examples of reflectance estimated spectra (380–750 nm) of Control corals and corals exposed to drill cuttings, barite and bentonite. X-axis scale 400–800 nm. All spectra shown are from coral polyp/calice.
(PDF)

S4 Fig Identification of a false annotation of coral 5 in the drill cutting control experiment with Hypix. (PDF)

S5 Fig Histogram of the differences between observers for all subjective ratings of all subjects. (PDF)
