# Supplementary material for: Exploring time series of hyperspectral images for cold water coral stress response analysis
Source: PLoS One. 2022 Aug 8;17(8):e0272408. doi: 10.1371/journal.pone.0272408 (PMC9359567; doi:10.1371/journal.pone.0272408)
Supplement: S1 Table — (PDF) [file pone.0272408.s001.pdf]

	Concentration	Time	Coral0	Coral1	Coral2	Coral3	Coral4
Control	0	T0.5	1	1	1	1	1
Control	0	T1	2	1	1	1	1
Control	0	T2	1	1	1	1	1
Barite	30	T0.5	5	3	3	1	2
Barite	30	T1	5	4	4	1	2
Barite	30	T2	5	4	3	1	2
Barite	50	T0.5	5	1	4	2	2
Barite	50	T1	5	1	4	2	1
Barite	50	T2	5	1	4	3	3
Barite	100	T0.5	1	1	1	2	3
Barite	100	T1	1	1	1	2	2
Barite	100	T2	1	1	2	2	2
Bentonite	30	T0.5	3	1	4	1	1
Bentonite	30	T1	2	1	4	1	1
Bentonite	30	T2	2	1	4	1	2
Bentonite	50	T0.5	4	1	3	2	2
Bentonite	50	T1	4	1	3	2	2
Bentonite	50	T2	4	1	2	2	2
Bentonite	100	T0.5	5	4	4	1	2
Bentonite	100	T1	5	3	4	1	3
Bentonite	100	T2	5	3	3	1	3
Control	0	T0.5	2	1	1	1	1
Control	0	T1	2	1	1	1	1
Control	0	T2	1	1	1	1	1
Barite	30	T0.5	5	3	3	1	1
Barite	30	T1	5	3	3	1	1
Barite	30	T2	5	3	3	1	1
Barite	50	T0.5	5	1	4	2	2
Barite	50	T1	5	1	4	2	2
Barite	50	T2	5	1	4	2	2
Barite	100	T0.5	2	1	2	2	2
Barite	100	T1	2	1	2	2	2
Barite	100	T2	1	1	2	2	2
Bentonite	30	T0.5	3	1	4	1	2
Bentonite	30	T1	3	1	4	1	2
Bentonite	30	T2	3	1	4	1	2
Bentonite	50	T0.5	4	1	3	2	2
Bentonite	50	T1	4	1	3	2	2
Bentonite	50	T2	4	1	3	2	2
Bentonite	100	T0.5	5	3	3	1	3
Bentonite	100	T1	5	3	3	1	4
Bentonite	100	T2	4	3	3	1	4
Control	0	T0.5	2	2	1	1	1
Control	0	T1	2	2	1	1	1

Control	0	T2	1	2	1	1	1
Barite	30	T0.5	5	3	3	1	1
Barite	30	T1	5	3	3	1	1
Barite	30	T2	5	3	3	1	1
Barite	50	T0.5	5	1	4	3	3
Barite	50	T1	5	1	4	3	3
Barite	50	T2	5	1	4	3	3
Barite	100	T0.5	3	2	2	4	4
Barite	100	T1	3	2	2	4	4
Barite	100	T2	3	2	3	4	4
Bentonite	30	T0.5	3	2	5	2	2
Bentonite	30	T1	3	2	5	2	2
Bentonite	30	T2	3	2	5	2	2
Bentonite	50	T0.5	5	1	5	3	3
Bentonite	50	T1	5	1	5	3	3
Bentonite	50	T2	5	1	5	3	3
Bentonite	100	T0.5	5	4	5	2	5
Bentonite	100	T1	5	4	5	2	5
Bentonite	100	T2	5	4	5	2	5

S1 Table: Individual subjective rating results for Bentonite and Barite experiments.
