# Supplementary material for: Exploring time series of hyperspectral images for cold water coral stress response analysis
Source: PLoS One. 2022 Aug 8;17(8):e0272408. doi: 10.1371/journal.pone.0272408 (PMC9359567; doi:10.1371/journal.pone.0272408)
Supplement: S2 Table — (PDF) [file pone.0272408.s002.pdf]

	Concentration	Time	Coral0	Coral1	Coral2	Coral3	Coral4	Coral5
Control	0	T0.5	1	1	1	2	1	4
Control	0	T1	1	1	2	2	1	4
Control	0	T2	1	1	1	2	1	5
Drill Cutting	10	T0.5	1	1	1	2	2	2
Drill Cutting	10	T1	1	1	1	2	1	1
Drill Cutting	10	T2	1	1	2	1	3	1
Drill Cutting	30	T0.5	2	2	2	2	1	2
Drill Cutting	30	T1	3	2	2	3	3	2
Drill Cutting	30	T2	3	2	2	3	2	2
Drill Cutting	50	T0.5	2	3	3	4	1	3
Drill Cutting	50	T1	2	2	1	4	2	3
Drill Cutting	50	T2	1	3	1	4	2	1
Drill Cutting	100	T0.5	2	2	1	4	2	2
Drill Cutting	100	T1	2	3	2	4	2	2
Drill Cutting	100	T2	4	3	2	3	2	3
Control	0	T0.5	1	1	1	2	1	4
Control	0	T1	1	1	2	2	1	4
Control	0	T2	1	1	1	2	1	5
Drill Cutting	10	T0.5	1	1	1	2	2	2
Drill Cutting	10	T1	1	1	1	2	1	1
Drill Cutting	10	T2	1	1	2	1	3	1
Drill Cutting	30	T0.5	2	2	2	2	1	2
Drill Cutting	30	T1	3	2	2	3	3	2
Drill Cutting	30	T2	3	2	2	3	2	2
Drill Cutting	50	T0.5	2	3	3	4	1	3
Drill Cutting	50	T1	2	2	1	4	2	3
Drill Cutting	50	T2	1	3	1	4	2	1
Drill Cutting	100	T0.5	2	2	1	4	2	2
Drill Cutting	100	T1	2	3	2	4	2	2
Drill Cutting	100	T2	4	3	2	3	2	3
Control	0	T0.5	1	2	1	4	2	4
Control	0	T1	1	2	1	2	2	4
Control	0	T2	1	2	1	2	2	5
Drill Cutting	10	T0.5	2	2	1	3	3	3
Drill Cutting	10	T1	2	2	1	2	1	2
Drill Cutting	10	T2	2	2	1	2	1	2
Drill Cutting	30	T0.5	2	3	2	2	2	2
Drill Cutting	30	T1	4	3	1	3	2	2
Drill Cutting	30	T2	4	3	1	3	2	2
Drill Cutting	50	T0.5	2	2	3	3	1	3
Drill Cutting	50	T1	2	2	2	3	1	3
Drill Cutting	50	T2	2	2	2	3	1	2
Drill Cutting	100	T0.5	3	3	2	3	2	2
Drill Cutting	100	T1	3	3	2	2	2	2
Drill Cutting	100	T2	3	3	2	2	2	2

S2 Table: Individual subjective rating results for drill cutting experiments.
