# Supplementary material for: Exploring time series of hyperspectral images for cold water coral stress response analysis
Source: PLoS One. 2022 Aug 8;17(8):e0272408. doi: 10.1371/journal.pone.0272408 (PMC9359567; doi:10.1371/journal.pone.0272408)
Supplement: S1 Text — (PDF) [file pone.0272408.s003.pdf]

S6 Text: Extraction of spectral signatures for reflectance estimation

Although the main analysis was carried out on radiance hyperspectral image data, reflectance estimation was done for selected coral pixels (ROI). The ROIs were manually extracted from the hyperspectral image using the software ENVI 5.4. Pixels from branches and calices were stored separately. Calice regions mainly consisted of pixels from the polyp, the living soft tissue of coral. The ROI was tagged with ID of the sample, color morph (white or orange), anatomy (branch or calice), time of recording (T0, T0.5, T1 or T2) and exposure treatment.

Reflectance conversion In order to obtain a measure of the true colour of the corals (e.g. reflectance or spectral signature), the attenuation of water was compensated for. This was done based on principles described by Letnes et al. (2019). This method for calculating reflectance of the coral regions uses the known reflectance of a reference plate. A Spectralon diffuse reflectance standard (Lapsphere Inc) and a grey polyethylene reference plate roughened with a 500 grit size sandpaper was measured with an Ocean Optics Flame spectrometer in air. The measured spectra of the Spectralon (Ispec) was compared with its calibrated reflectance values (Rspec) and hence, the conversion factor A was found.

$$A = \frac{Ispec(\lambda)}{(Rspec(\lambda))}$$

The conversion factor A and spectrometer measured spectra of the polyethylene reference plate (Iref) enabled calculation of the reflectance of the reference plate (Rref).

$$Rref(\lambda) = \frac{Iref(\lambda)}{A}$$

The reference plate was placed in aquarium immediately behind the corals as illustrated in Figure S2 and S3. As the corals were placed in the diagonal plane, they had different distances to the sensor. The distance was measured using a ruler. To compensate for the difference in attenuation due to optical path length variation between the corals, each coral sample got assigned an individual selection of reference pixels taken adjacent to the coral. The minor distance between reference and coral was neglected. The conversion factor between UHI measured spectra and reflectance could such be calculated for all spatial pixels in the slit at any distance from target.

$$A(x, y) = \frac{Iref(x, y, \lambda)}{Rref(\lambda)}$$

The reflectance of the coral sample ROIs could then be calculated.

$$Rcoral(\lambda) = \frac{Icoral(x, y, \lambda)}{A(x, y)}$$

Note that while the reference plate has a diffuse reflection profile, the different coral regions on the other hand might have significantly more specular components. This can show up in the calculated reflectance as regions having a reflectance greater than 1. This is due to more of the light being reflected into a smaller angle compared to the reference plate resulting in higher measured intensity. The occurrence of these regions will be dependent on both the reflection profile of the coral, and the relative illumination angle from the light-source onto the coral surface in relation to the UHI camera. This difference in specular components is not compensated for during the analysis.
