# Supplementary material for: Exploring time series of hyperspectral images for cold water coral stress response analysis
Source: PLoS One. 2022 Aug 8;17(8):e0272408. doi: 10.1371/journal.pone.0272408 (PMC9359567; doi:10.1371/journal.pone.0272408)
Supplement: S1 Fig — Left: Photo of corals (white and orange) and polyethylene reference plate inside aquarium. Right: Sketch showing position of time lapse camera and underwater hyperspectral imager (UHI) outside the aquarium with coral nubbins. (PDF) [file pone.0272408.s004.pdf]

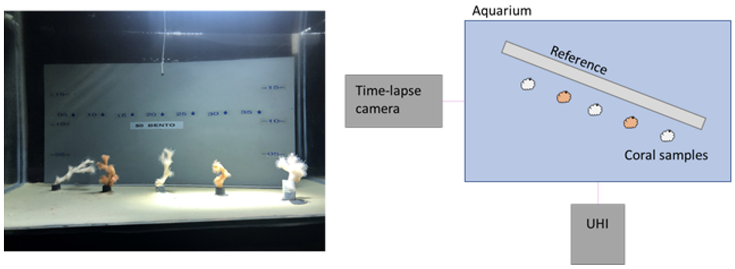

S3 Figure: Laboratory Set-up: Aquarium setup Left: Photo of corals (white and orange) and polyethylene reference plate inside aquarium. Right: Sketch showing position of time lapse camera and underwater hyperspectral imager (UHI) outside the aquarium with coral nubbins.
