# Supplementary material for: Exploring time series of hyperspectral images for cold water coral stress response analysis
Source: PLoS One. 2022 Aug 8;17(8):e0272408. doi: 10.1371/journal.pone.0272408 (PMC9359567; doi:10.1371/journal.pone.0272408)
Supplement: S2 Fig — The hyperspectral imager was placed on a scanning rig outside the aquarium, scanning through the glass. (PDF) [file pone.0272408.s005.pdf]

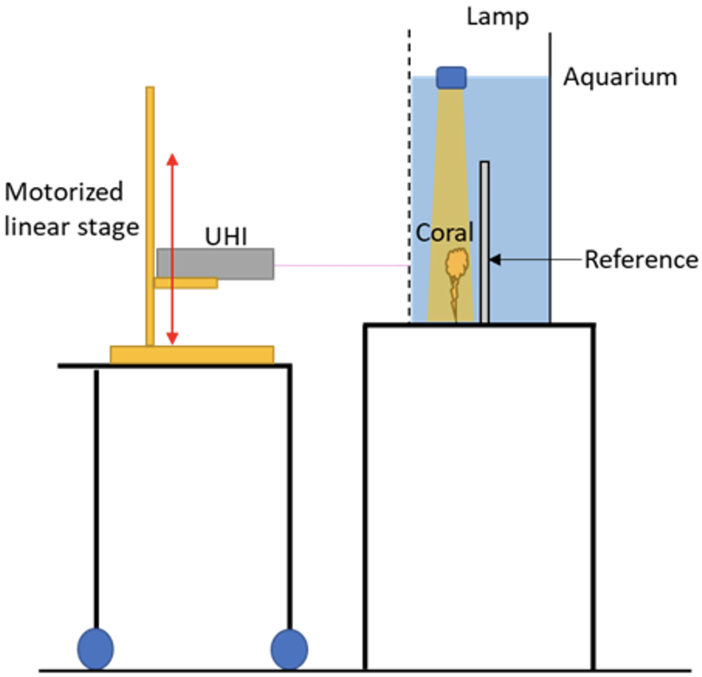

S4 Figure: Laboratory set-up: Setup for acquisition of hyperspectral images Coral samples and a polyethylene reference plate were placed at the bottom of aquarium. The hyperspectral imager was placed on a scanning rig outside the aquarium, scanning through the glass.
