# Supplementary material for: Exploring time series of hyperspectral images for cold water coral stress response analysis
Source: PLoS One. 2022 Aug 8;17(8):e0272408. doi: 10.1371/journal.pone.0272408 (PMC9359567; doi:10.1371/journal.pone.0272408)
Supplement: S3 Fig — X-axis scale 400–800 nm. All spectra shown are from coral polyp/calice. (PDF) [file pone.0272408.s006.pdf]

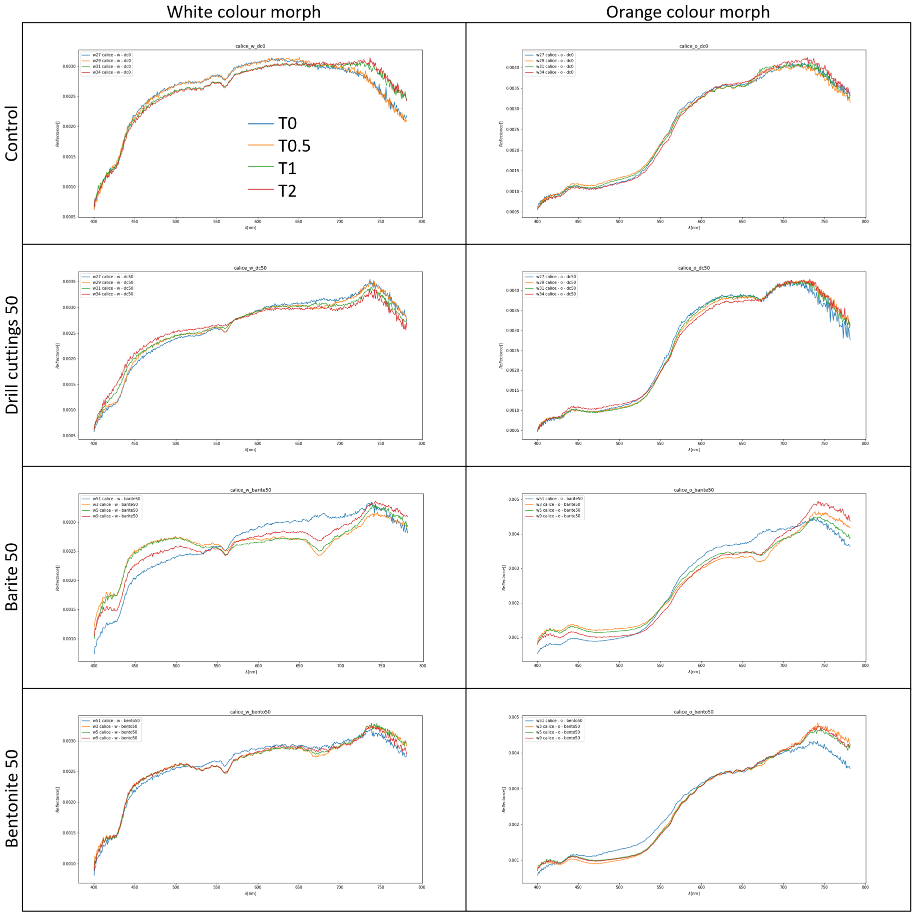

S5 Figure: Examples of reflectance estimated spectra (380 – 750 nm) of Control corals and corals exposed to drill cuttings, barite and bentonite. X-axis scale 400 – 800 nm. All spectra shown are from coral polyp/calice.
