# Supplementary material for: Exploring time series of hyperspectral images for cold water coral stress response analysis
Source: PLoS One. 2022 Aug 8;17(8):e0272408. doi: 10.1371/journal.pone.0272408 (PMC9359567; doi:10.1371/journal.pone.0272408)
Supplement: S4 Fig — (PDF) [file pone.0272408.s007.pdf]

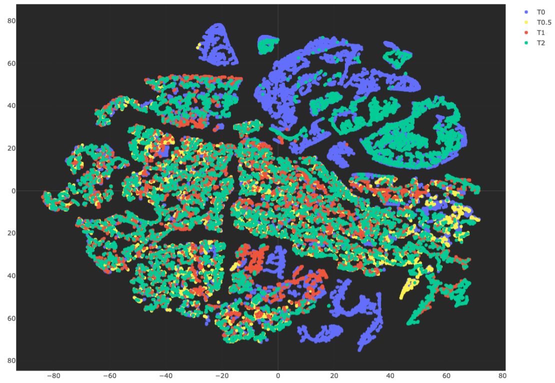

(a) Original scatter plot

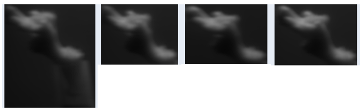

(b) One can clearly spot the much larger image for T0

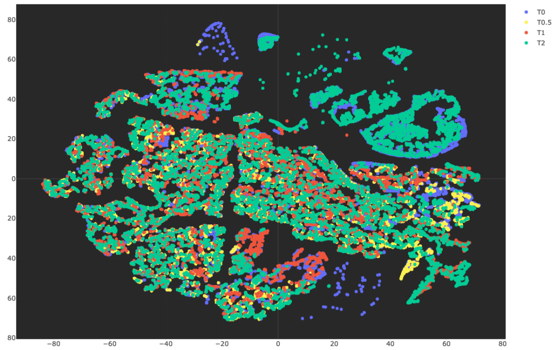

(c) The scatter plot after selecting the correct data for T0 matching the other timepoints.

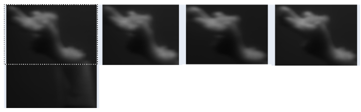

(d) The selection frame for T0.

S7 Figure: Identification of a false annotation of coral 5 in the drill cutting control experiment with Hypix.
