# Supplementary material for: Exploring time series of hyperspectral images for cold water coral stress response analysis
Source: PLoS One. 2022 Aug 8;17(8):e0272408. doi: 10.1371/journal.pone.0272408 (PMC9359567; doi:10.1371/journal.pone.0272408)
Supplement: S5 Fig — (PDF) [file pone.0272408.s008.pdf]

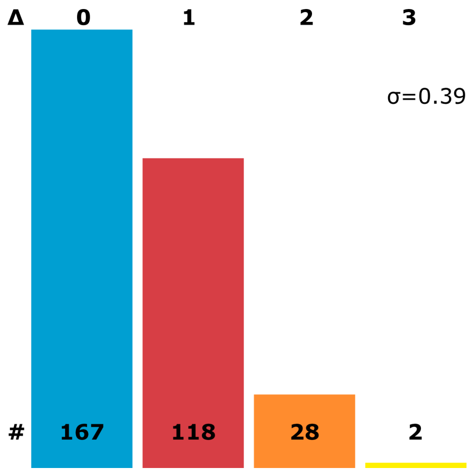

S8 Figure: Histogram of the differences between observers for all subjective ratings of all subjects.
